# Correlation of the Dietary Protein Intake between Those Estimated from a Short Protein Food-Recall Questionnaire and from 24-Hour Urinary Urea-Nitrogen Excretion in Stages 3-4 Chronic Kidney Disease Patients

**DOI:** 10.1155/2023/9713045

**Published:** 2023-11-22

**Authors:** Teerawat Thanachayanont, Methee Chanpitakkul, Akhathai Saetie, Salyaveth Lekagul, Kriang Tungsanga

**Affiliations:** ^1^Bhumirajanagarindra Kidney Institute, 8/99 Phayathai Rd, Khwaeng Thung Phaya Thai, Khet Ratchathewi, Bangkok 10400, Thailand; ^2^Department of Medicine, King Chulalongkorn Memorial Hospital, Chulalongkorn University, Bangkok, Thailand

## Abstract

**Introduction:**

High protein intake may accelerate progression of chronic kidney disease (CKD). Estimation of dietary protein intake (DPI) is indispensable for management of CKD, but to achieve optimum DPI is quite challenging in routine clinical practice. We recently studied a beneficial effect of utilizing integrated care on the management of CKD at the rural community level. In that study, we created a short protein food-recall questionnaire (S-PFRQ) as a working tool to estimate DPI of the CKD patients during home visit by community health personnel. Herein, we reported the initial evaluation of the reliability of S-PFRQ from our previous study.

**Objective:**

We compared the amount of DPI obtained from S-PFRQ with that obtained from protein-equivalent of total nitrogen appearance (PNA).

**Methods:**

In the previous ESCORT-2 study, 914 patients with CKD stage 3 or 4, who were living in the rural area of Thailand, were prospectively followed while receiving integrated care for 36 consecutive months. During home visits by community nurses from subdistrict health centers, dietary food recall was made, recorded in S-PFRQ, and DPI was obtained. Among these, sixty patients were randomly selected, and 24-h urine was collected for urinary urea-N and estimation of PNA. A correlation was made between DPI obtained from S-PFRQ and PNA.

**Results:**

The DPIs derived from S-PFRQ and PNA were 28.8 ± 14.8 and 39.26 ± 17.79 g/day, respectively. The mean difference and 95% CI between the 2 methods was −10.43 (−7.1 to −13.8) g/day, respectively (*P* < 0.001). Interclass correlation between these 2 methods was 0.24, *P* = 0.007. The difference between the 2 methods remained constant across different amounts of DPI.

**Conclusion:**

The DPI estimated from S-PFRQ significantly correlated to that from PNA. However, the S-PFRQ method yielded a DPI value which was about 10 g of protein or 25% less than the PNA method. Despite this amount of difference, this S-PFRQ is user-friendly and could be used during field work as an easy and simple tool for DPI estimation in resource-limiting condition.

## 1. Introduction

Chronic kidney disease (CKD) is a growing healthcare problem worldwide. It is estimated that about 800 million population on earth harbor CKD at any stage [1]. CKD is associated with increased risks of cardiovascular diseases and premature mortality. Moreover, CKD leads to poor quality of life of individuals and their families [2]. Expense and resource utilization incurred from CKD management is extremely high [3]. In Thailand, about 17% of the total population could have CKD stages 3–5 (nondialysis) [4]. Hemodialysis is the main renal replacement modality in Thailand and cost about 60 USD per session or about 7800 USD per patient per year [5]. Kidney replacement therapy expenditure was approximately as high as 3% of total health expenditure of Thailand [6]. At an early stage of the disease, CKD prevails not only at secondary- or tertiary-care hospitals but also in the rural community across the country. Dietary modification is recommended in the practice guideline for delaying progression of CKD [7]. Patients with CKD should have moderate to stringent restrictions on protein and salt consumption [8]. Previous studies reported that patients with CKD who had received consultation from dietitians had slower progression of CKD [9, 10]. However, accessibility to such dietary care is usually confined to developed countries or at major tertiary-care hospitals where qualified dieticians are abundantly available. In a recent cross-sectional survey study from United States of America, the rate of referral for medical nutritional therapy was only 50.7% [11]. In developing countries, counseling a dietician for patients with CKD is practically low [12]. Thus, it is mandatory to identify a simple and pragmatic way to estimate dietary protein intake (DPI) among patients with CKD in resource-limited countries.

At present, several alternative methods have been utilized as a tool for estimating DPI, namely, 3-day dietary recall, dietary food record, 24-h urine collection, and short food recall questionnaire [13, 14]. The first 2 methods require much patient's understanding and compliance with dietitian's instruction. Accuracy of the methods also relies on consistency of dietary ingredients of the food recipes. Thus, it might be subject to estimation error when it is applied to oriental foods, which commonly do not have exact portion of the ingredients. Moreover, an experienced dietitian is required to calculate the amount of nutrient intake. Collection of 24-h urine, for measuring urine urea-nitrogen (urea-N) and estimation of protein-equivalent of total nitrogen appearance (PNA), seems to be a more accurate method and less dependent on specialized personnel [15]. However, it is inconvenient for patients, subject to collection error, and not practical to use in a primary care setting.

In our previous studies among patients with CKD stages 3-4 who were residing in rural communities of Thailand, we could demonstrate that our model of an integrated care for CKD conducted by healthcare personnel available in the rural healthcare setting (i.e., consisting of visiting a multidisciplinary care team at the district hospital, in conjunction with receiving home visit by community nurses and village health volunteers from the subdistrict health centers) could be effective in delaying CKD progression [16, 17]. In the latter study [17], the community nurses from subdistrict health centers were assigned to use a short protein food recall questionnaire (S-PFRQ) as a simple tool for estimating an amount of protein consumed by each patient to provide appropriate nutritional counseling. In this communication, we intended to correlate the results of DPI obtained from S-PFRQ and PNA.

## 2. Methods

The ESCORT-2 study protocol was approved by the Ethics Committee of Institutional Review Board, Ministry of Public Health, Thailand, and registered with https://www.clinicaltrials.in.th (TCTR-20160614001). Details of the study had been described elsewhere [17]. Importantly, patients who had heavy proteinuria (more than 2+ of proteinuria on urine dipstick or 3.5 g per day) were not eligible for that study. In that communication, patients with stage 3 or 4 CKD were taken care of by a multidisciplinary care team at district hospitals. In addition, they also received home visits twice a year from a community care network team (CCNT). The CCNT comprised a community nurse from subdistrict health center and a village health volunteer who was responsible for primary health care of the corresponding households. CCNT advised the patients on how to conduct healthy lifestyles with respect to CKD, interviewed about their daily activities, their consumption of salty or high-protein foods, and other unhealthy behavior. Of all patients who were enrolled in the ESCORT-2 study, patients with their research identification numbers ended with 0, 15, 30, 45, 60, etc., were randomly selected for this study. If any of these patients were not available, those who had a research identification number next in order were invited instead. Written informed consent was obtained prior to the enrollment.

### 2.1. Development of a Short Protein Food Recall Questionnaire (S-PFRQ)

In the beginning, an interview was conducted with nutritionists who were working at the local district hospitals. Eight food items containing high protein, which were commonly consumed by rural Thais, were identified. These included egg white, whole egg, mackerel, meat, processed meat, seafood, meatball, and milk. For a patient's convenience, the unit of expression of each food item was a common unit which was used daily by and easily understandable among villagers, e.g., a number of eggs or fish, a number of soup spoons, etc. Prior to initiation of the trial, members of CCNT were trained by a renal dietician on how to interview patients and how to record on S-PFRQ. A 7-day period was set as a time interval for food intake recall. Records on S-FPRQ were made every 6 months during home visit throughout the 3-year study period of the ESCORT-2 study.

### 2.2. Estimation of DPI Based on S-PFRQ

**Figure 1:**
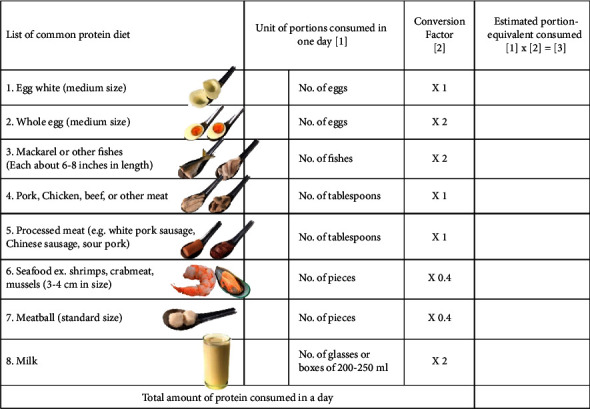
Short-protein food recall questionnaire English version. The first column is the list of 8 groups of common protein diet, the 2^nd^ column is the portion size, the 3^rd^ column is the conversion factor for each food group, and the last column is the estimated equivalent portion of protein intake.

The total amount of food items consumed during a 7-day recall was converted from the local units as such to a standard protein portion, i.e., one portion of protein intake or about 30 g of meat contains about 7 grams of protein [18]. Then, this amount was averaged to a daily DPI. The details of short protein food recall questionnaire (S-PFRQ) are shown in Figure 1.

### 2.3. Calculation of Dietary Protein Intake from the 24-h Urine Collection

Throughout the ESCORT-2 study, the enrolled cases in this study were asked to collect 24-h urine, while staying at home, and every 6 months thereafter. The urine specimens were sent to the Department of Laboratory Medicine, Kamphaeng Phet Hospital, Kamphaeng Phet Province, for analysis of creatinine, and urea-N. Creatinine was assayed with an enzymatic method. Calculation of protein-equivalent of total nitrogen appearance (PNA), which reflected daily protein intake, was calculated with Mitch's formula. [PNA = 6.25 × [UUN + (0.031 × ideal body weight)], where UUN stood for 24-h urinary urea-N excretion] [15].

### 2.4. Statistical Analysis

Continuous variables were expressed as the mean ± standard deviation [SD]. Independent *t*-test and ANOVA were performed for mean comparison between 2 categories and 3 categories variables, respectively. Pair *t*-test was used to compare the amount of DPI obtained from these 2 methods. Intraclass correlation coefficient [ICC] was used to compare reliability of DPI which was derived from these 2 methods. The Bland–Altman plot was made to analyze the agreement of these 2 methods. The calculation was made with SPSS software, version 23.

## 3. Results

**Figure 2:**
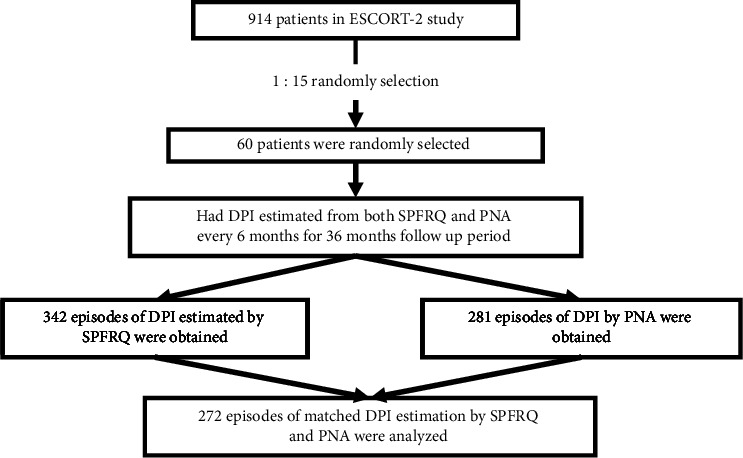
Study flow chart.

Among 914 patients enrolled in the ESCORT-2 study, 60 cases were eligible for the current study (Figure 2). Altogether, concurrent S-PFRQ interview and PNA estimation could be achieved in 272 episodes over the 36-month follow-up period.

The mean age was 62.0 ± 7 years, with a male to female ratio of 1 : 2.5. Twenty-one, 28, and 11 cases were classified as CKD stages 3A, 3B, and 4B, respectively. Diabetes, hypertension, and cardiovascular diseases were present in 52%, 97%, and 3% of cases, respectively. The baseline eGFR was 40.3 ± 9.73 mL/min/1.73 m^2^ body surface area. The BMI was 25.1 ± 2.83 kg/m^2^. The baseline serum albumin was 4.27 ± 0.41 g/dL. Fifty-eight percent of participants had positive proteinuria detected by urine dipstick. The 24-h urine volume was 1581 ± 612 ml (Table 1).

**Figure 3:**
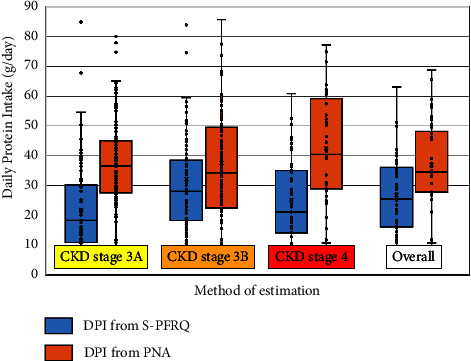
Box and Whisker plot shows comparison of mean DPI estimation from the 2 methods in different stages of CKD.

Overall, DPIs estimated from S-PFRQ and PNA were 28.8 ± 14.8 and 39.26 ± 17.79 g/day, respectively. These were equal to 0.46 ± 0.03 and 0.59 ± 0.09 g/kg/day of protein intake estimated from S-PFRQ and PNA, respectively. The overall mean differences of DPI estimation between these 2 methods (S-PFRQ–PNA) were −10.43 g protein/day (95% CI −7.1 to −13.8). The mean differences of DPI estimation from the 2 methods in patients with stage 3A, 3B, and 4 CKD were −18.35, −7.18, and −16.83 g protein/day, respectively (Table 2 and Figure 3). The mean difference of DPI estimation was statistically lower in the stage 3B patient group than stage 3A and 4 patient groups. The mean difference of DPI estimation in patients with diabetes was −12.26 g protein/day which was higher than patients without diabetes but not statistically significant. Also, patients with proteinuria tended to have higher difference of DPI estimation than patients without proteinuria (Table 3).

**Figure 4:**
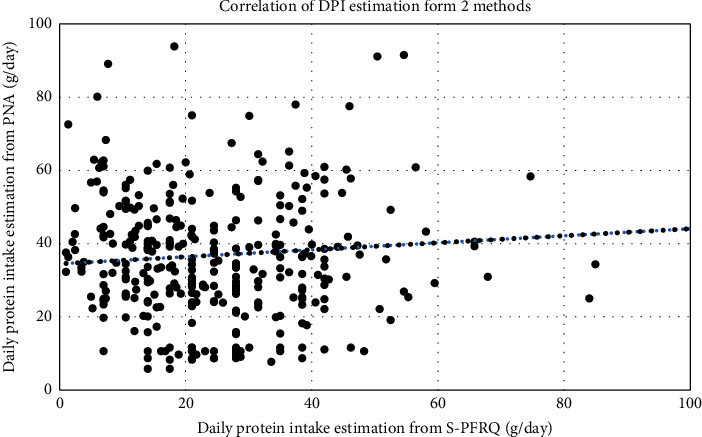
Graph represents correlation of DPI estimation from 2 methods; *Y*-axis represents DPI calculated from 24-h urine collection, *X*-axis represents DPI estimation from S-PFRQ, unit in g per day.

**Figure 5:**
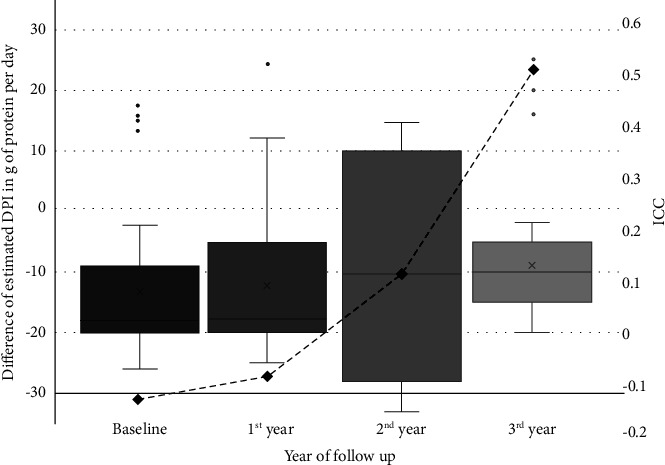
Difference of estimated DPI (Bar) and ICC (dotted line) between 2 methods over study time period.

The Pearson correlation coefficient of DPIs estimated from these 2 methods was 0.126 (*P* = 0.021) (Figure 4). The mean differences of DPI estimation from the 2 methods were −13.28 g protein/day at the baseline and subsequently were −12.21, −9.93, and −8.98 in first, second, and third years, respectively. The intraclass correlation was −0.12, −0.09, 0.12, and 0.51 at the baseline, at the first, second, and third years, respectively (Figure 5). Overall, intraclass correlation (ICC) between these 2 methods of DPI estimation was 0.24 (95% CI = 0.041–0.392, *P* = 0.007).

**Figure 6:**
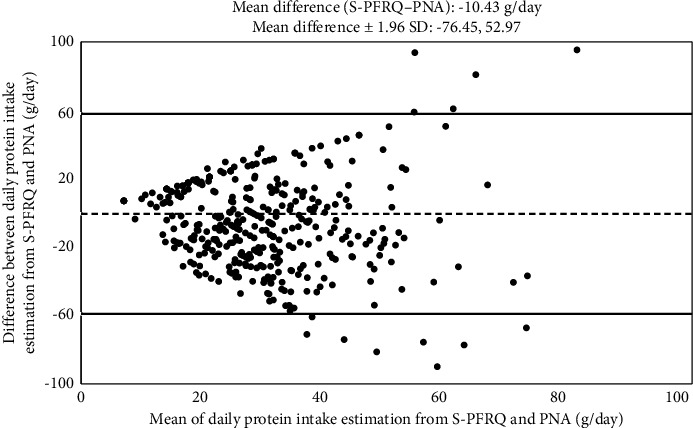
Bland–Altman plots between the DPI estimated from PNA and S-PFRQ.

The Bland–Altman plot was done to elucidate bias and limit of agreement of these 2 methods of DPI estimation as shown in Figure 6. About ninety-five percent of the values lie within the limit of agreement. The difference of estimated DPIs was relatively stable across a broad range of the mean DPIs of the 2 methods.

## 4. Discussion

CKD is now a significant health problem in both developed and developing countries. In the latter country group, limited access to kidney replacement therapy due to several factors signifies that delaying kidney disease progression is of prime importance. Among these disease-modifying factors, protein restriction is one of the key recommendations [7]. The proper amount of DPI recommended in clinical practice guidelines is quite consistent [7, 19, 20]. Dietary assessment tools are important for improving patient adherence to nutritional advice. However, identifying a practical, sustainable, and user-friendly tool for accessing DPI has remained a problematic issue. In this study, we could demonstrate that first, the DPI obtained from S-PFRQ correlated reasonably well with that from the PNA method, with a mean difference of only −10.43 g of protein per day, or DPI estimated from S-PFRQ was about 25% lower than DPI estimated from PNA (Figure 6). Second, ICC of DPI estimation from these 2 methods progressively increased over time. Lastly, up to 95% of the values lie within the limit of agreement indicating that this difference is consistent across a broad range of the amount of DPI (Figure 6).

Estimation of DPI with the S-PFRQ method could be lower than the PNA method due to several possible explanations including imperfect recall leading to underreporting of protein intake and consumption of protein from other foods not listed in the questionnaire. Food recall is a simple and convenient dietary intake assessment method. Yet, it relies on patient's memory which could be imperfect particularly in our study participants who are aging and undereducated population. A previous study conducted in non-CKD patients showed that daily protein intake estimation from 24-hour food recall was lower than urinary nitrogen excretion by 25.5% [21]. A study done in CKD patients also showed that the food record method underestimates protein intake compared to 24-hour urine collection [22, 23]. Our findings are in accord with these publications. Besides patients' memory, interviewer skill and experience are also important. ICC between these 2 daily protein estimation methods is increased over the time of follow-up suggesting that the skill and experience of healthcare providers in interviewing food recall had been improving over time, resulting in more accurate results. Some participants might tend to underreport protein intake to show that they have good compliance with healthcare worker nutritional counseling. Studies done in dialysis and nondialysis CKD patients showed that underreporting energy intake was found in more than 50% of patients [24, 25]. The eight food items enlisted in the S-PFRQ were only the most consumed among villages. Certainly, there could have been many other kinds of food, which might contain a considerable amount of protein, and might not have been included in the questionnaire. Thus, part of food protein might have been omitted from the calculation. Nevertheless, the difference of DPI values obtained from the two methods of estimation is only 10 g of protein per day. Moreover, this difference as such was stable across a wide range of dietary protein consumption. It could reflect consistent accuracy of the S-PFRQ.

Proteinuria is another factor that could affect estimation of protein intake by using the PNA method. In this study, we did not estimate proteinuria quantitatively. Thus, mean differences of DPI estimation from 2 methods could be higher than our findings. However, in this study, patients with heavy proteinuria were not eligible for participation [17]. Thus, the effect of proteinuria on our results should be minimal. Furthermore, patients with proteinuria tended to have higher differences of DPI but not reaching statistical significance (Table 3). Whether the presence of proteinuria could exert a clinically significant effect on DPI estimation needs further study.

There are several limitations in our study. Due to 24-hour urine collection technical difficulties, we therefore randomly selected only 60 patients for initial evaluation of our food recall questionnaire performance. Secondly, proteinuria was assessed with the urine dipstick test. Proteinuria was therefore not quantified in the estimated daily protein intake from the PNA method. Lastly, our food recall questionnaire was designed for a 7-day period which might be too long period of time to recall. However, a 7-day period is intended to reduce selection bias and daily protein intake is supposed to be an average of 7-day period.

Dietary protein modification would be effective for delaying CKD progression if it is implemented at an early stage of the disease when the patient is symptom-free. Having this simple questionnaire tool in hands, it will be convenient for primary health personnel, such as community nurses at subdistrict health office or the other, to give advice or instruct the patients to avoid consuming high amounts of dietary protein. Our S-PFRQ could be an example of a simple, but moderately efficient dietary assessment tool that could be adapted to use in other resource-limiting countries. It would create better healthcare professional-patient communication and promote adherence to low protein diet counseling [26, 27]. Nevertheless, S-PFRQ needs to be adjusted according to different patterns of foods of each country. In addition, a dietary intake survey needs to be done prior to enlisting high protein food items into the questionnaire. A commonly used unit and a picture of each food item should also be utilized in the questionnaire.

We hope that implementing this simple and friendly used tool in primary care settings would enable local health care personnel to be a meaningful driving force on delaying CKD progression at an early stage as expected.

## Figures and Tables

**Table 1:** Participants' demographic and biochemical data.

Parameters	Values
Age, years (mean ± SD)	62 ± 7.8
Female, case (%)	43 (71.6%)
Education level, case (%)	
Uneducated	9 (15%)
Elementary school	51 (85%)
Diabetic kidney disease, case (%)	31 (51.66%)
Hypertension, case (%)	58 (96.66%)
Cardiovascular disease, case (%)	2 (3.33%)
Stage of CKD,	
3A cases (%)	21 (35%)
3B cases (%)	28 (46.7%)
4 cases (%)	11 (18.3%)
Body weight, kg (mean ± SD)	62.0 ± 4.24
BMI, kg/m^2^ (mean ± SD)	25.1 ± 2.83
eGFR, ml per min per 1.73 m^2^ (mean ± SD)	40.3 ± 9.73
Serum creatinine, mg/dl (mean ± SD)	1.58 ± 0.38
Serum albumin, g/dl (mean ± SD)	4.27 ± 0.41
LDL cholesterol, mg/dl (mean ± SD)	102 ± 29.5
Proteinuria status	
No proteinuria	25 (41.7%)
Proteinuria positive	35 (58.3%)
Mean urine volume, ml (mean ± SD)	1581 ± 612

**Table 2:** *T*-test comparison of DPI estimation from the 2 methods in different stages of CKD.

Stage of CKD	Number of study participants	Estimated DPI		Difference: mean (95% CI)	*P* value
		S-PFRQ	PNA		
3A	21	21.22 ± 10.26	39.58 ± 13.62	−18.35 (−25.21 to −11.49)	0.0001
3B	28	33.43 ± 12.14	40.62 ± 18.70	−7.18 (−17.87 to 3.50)	0.175
4	11	25.14 ± 13.51	41.49 ± 18.10	−16.83 (−20.57 to 10.3)	0.357

CKD = chronic kidney disease, DPI = daily protein intake, PNA = protein-equivalent of total nitrogen appearance, S-PFRQ = short-protein Food Recall Questionnaire. Mean difference = DPI estimation from S-PFRQ minus DPI estimation from PNA.

**Table 3:** Estimated daily protein intake (DPI) by various clinical factors.

Factors	Number of study participants	Estimated DPI		Mean difference		*P* value (comparison mean difference between categories in each group of clinical factors)
		S-PFRQ	PNA			
*Causes of CKD*						
Non-DKD	29	30.83 ± 17.68	42.59 ± 19.96		−11.76	0.172
DKD	31	26.22 ± 9.52	38.82 ± 10.35		−12.60	

*Stages of CKD*						
3A	21	21.22 ± 10.26	39.58 ± 13.62		−18.35	0.0001 [3A vs 3B]
3B	28	33.43 ± 12.14	40.62 ± 18.70		−7.18	0.029 [3B vs 4]
4	11	25.14 ± 13.51	41.49 ± 18.10		−16.83	0.919 [3A vs 4]

*Proteinuria status*						
No proteinuria	25	26.70 ± 10.13	36.86 ± 15.18		−10.19	0.094
Positive proteinuria	35	30.70 ± 17.98	44.29 ± 15.73		−13.84	

CKD = chronic kidney disease, DPI = daily protein intake, PNA = protein-equivalent of total nitrogen appearance, S-PFRQ = short-protein food recall questionnaire. Mean difference = DPI estimation from S-PFRQ minus DPI estimation from PNA.

## Data Availability

The data used to support the findings of this study are available from the corresponding author upon request.
